# Whole Nervous System Expression of Glutamate Receptors Reveals Distinct Receptor Roles in Sensorimotor Circuits

**DOI:** 10.1523/ENEURO.0306-24.2024

**Published:** 2024-09-18

**Authors:** Cezar Borba, Matthew J. Kourakis, Yishen Miao, Bharath Guduri, Jianan Deng, William C. Smith

**Affiliations:** ^1^Department of Molecular, Cell and Developmental Biology, University of California, Santa Barbara, California 93106; ^2^Neuroscience Research Institute, University of California, Santa Barbara, California 93106

**Keywords:** *Ciona*, connectome, evolution, glutamate receptor, invertebrate

## Abstract

The goal of connectomics is to reveal the links between neural circuits and behavior. Larvae of the primitive chordate *Ciona* are well-suited to make contributions in this area. In addition to having a described connectome, *Ciona* larvae have a range of readily quantified behaviors. Moreover, the small number of neurons in the larval CNS (∼180) holds the promise of a comprehensive characterization of individual neurons. We present single-neuron predictions for glutamate receptor (GlutR) expression based on in situ hybridization. Included are both ionotropic receptors (AMPA, NMDA, and kainate) and metabotropic receptors. The predicted glutamate receptor expression dataset is discussed in the context of known circuits driving behaviors such as phototaxis, mechanosensation, and looming shadow response. The predicted expression of AMPA and NMDA receptors may help resolve issues regarding the co-production of GABA and glutamate by a subset of photoreceptors. The targets of these photoreceptors in the midbrain appear to express NMDA receptors, but not AMPA receptors. This is in agreement with previous results indicating that GABA is the primary neurotransmitter from the photoreceptors evoking a swimming response through a disinhibition mechanism and that glutamate may, therefore, have only a modulatory action in this circuit. Other findings reported here are more unexpected. For example, many of the targets of glutamatergic epidermal sensory neurons (ESNs) do not express any of the ionotropic receptors, yet the ESNs themselves express metabotropic receptors. Thus, we speculate that their production of glutamate may be for communication with neighboring ESNs, rather than to their interneuron targets.

## Significance Statement

Simple invertebrates offer a tractable alternative to complex vertebrate brains, facilitating holistic understanding of brain function. One such invertebrate is the marine chordate *Ciona*, which has the benefit of a complete synaptic wiring diagram for its swimming larva. This “connectome” allowed identification of putative neural circuits driving defined behaviors. Fuller understanding of neural circuits, however, requires a description of the attributes of individual neurons. This study focuses on the excitatory neurotransmitter glutamate, which signals via a complex set of both ionotropic and metabotropic receptors. Here, we present a nervous system-wide prediction of GlutR expression in *Ciona* at the individual neuron level, considered in the context of neural circuits, with emphasis on how GlutR expression accounts for function of neural circuits.

## Introduction

**Figure 1.**
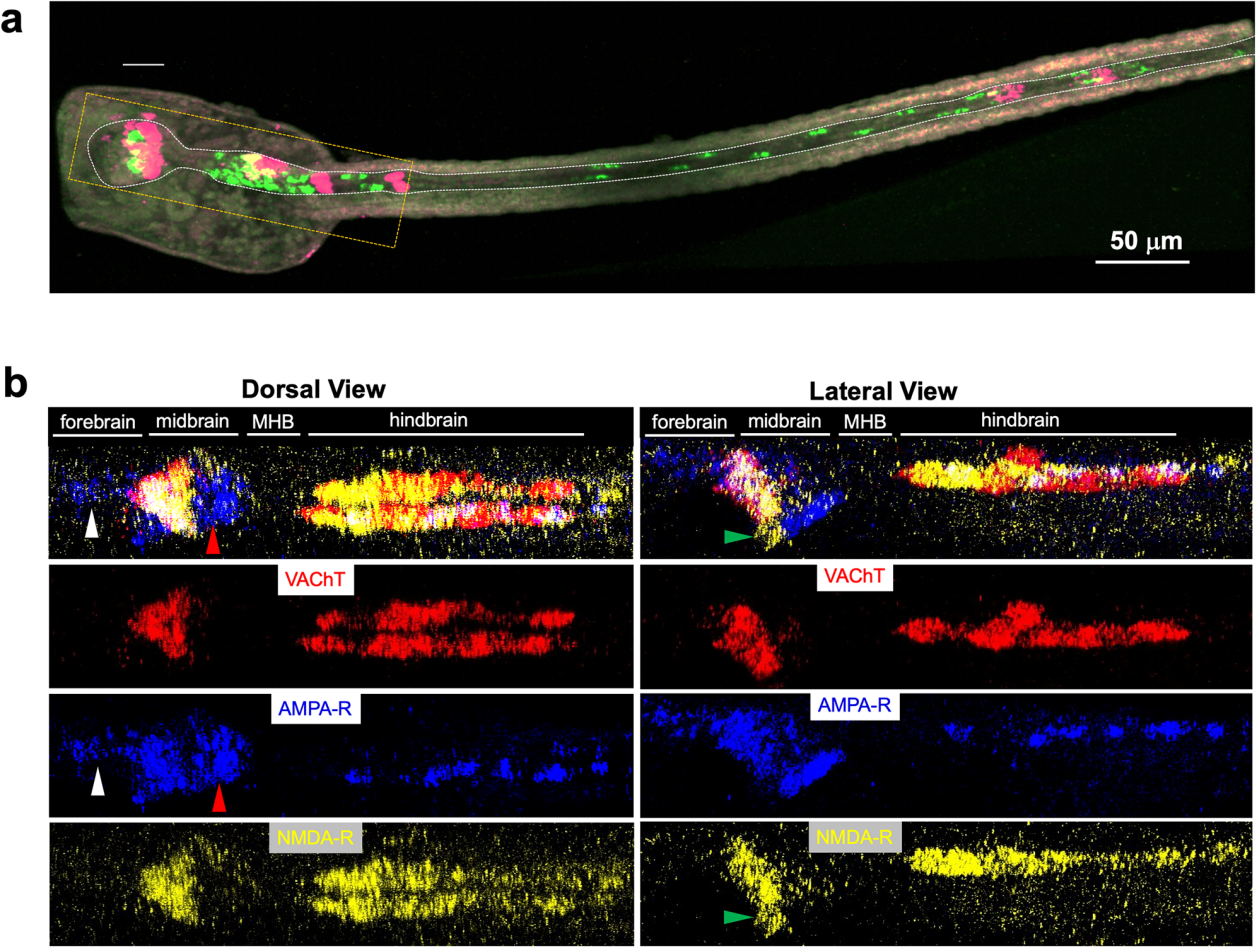
In situ hybridization of *C. robusta* larvae. ***a***, In situ hybridization of *C. robusta* larva for VAChT and VGAT. The major subdivisions of the central nervous system are labeled according to their vertebrate orthologs. The white dotted line outlines the central nervous system and the orange box outlines the approximate brain regions shown below in panel ***b***. ***b***, In situ hybridization of *C. robusta* larva for VAChT, AMPAR, and NMDAR. The top panels show the dorsal and lateral composite views of the three expression patterns. The three bottom right and left panels show the individual images that comprise the composite. The white and red arrowheads indicate the AMPAR^+^/NMDAR^−^ forebrain and posterior midbrain neurons, respectively. The green arrowheads indicate the NMDAR^+^/AMPAR^−^ neurons. The blue arrowhead indicates the asymmetric AMPAR expression (left side only); while the orange arrowheads show that NMDAR is symmetric in the left/right axis. All images are maximum-intensity *z*-projection images from confocal stacks. Anterior is to the left for all panels.

The tadpole larva of the invertebrate chordate *Ciona* is a highly tractable model for sensorimotor circuit analyses. Not only does the *Ciona* larval central nervous system (CNS) contain only ∼180 neurons, it is one of the few animals for which a complete synaptic connectome has been described (Ryan et al., 2016). Moreover, numerous studies have highlighted the conservation between the *Ciona* larval CNS and those of vertebrates [reviewed by Hudson (2016)]. At the anatomical level, the *Ciona* CNS is subdivided into domains showing homology to the vertebrate forebrain, midbrain, hindbrain, midbrain–hindbrain boundary (MHB), and spinal cord (Fig. 1*a*). These homologies are evident in *Ciona*'s developmental mechanisms, gene expression, and anatomy and, more recently, in neuron classification and synaptic connectivity (Wada et al., 1998; Hudson, 2016; Ryan et al., 2017; Borba et al., 2021). Early descriptions of larval tunicate nervous systems, often made before the above homologies were clear, led to the naming of these anatomical domains with names that do not reflect this homology (e.g., anterior sensory vesicle or anterior brain vesicle for the *Ciona* forebrain homolog, posterior sensory vesicle or posterior brain vesicle for the *Ciona* midbrain homolog, and visceral or motor ganglion for the hindbrain homolog). For the sake of clarity, and to make *Ciona* neurobiology accessible to a broader readership, we will henceforth refer to the *Ciona* CNS anatomical domains according to their vertebrate homologs.

The *Ciona* connectome dataset has been instrumental in identifying neural circuits that drive a number of larval behaviors, including negative phototaxis, negative gravitaxis, a touch response, and a looming shadow response (Ryan et al., 2016, 2018; Kourakis et al., 2019; Bostwick et al., 2020). The connectome, however, provides only a “bare-bones” view of the neural circuitry. A fuller understanding of the logic of neural circuits requires knowledge of the properties of the constituent neurons (e.g., neurotransmitter use and neurotransmitter receptor expression). A prediction of neurotransmitter use in the *Ciona* larval CNS is emerging through analysis of in situ hybridization patterns for markers of small-molecule neurotransmitters [e.g., vesicular acetylcholine transporter (VAChT) for cholinergic neurons, vesicular GABA transporter (VGAT) for GABAergic/glycinergic neurons, tyrosine hydroxylase (TH) for catecholaminergic neurons, and tryptophan hydroxylase (TPH) for serotonergic neurons; Moret et al., 2005; Pennati et al., 2007; Kourakis et al., 2019]. The low number of larval neurons and the largely stereotyped expression patterns facilitate the mapping of neurotransmitter use to the connectome (Kourakis et al., 2019). For example, all vesicular glutamate transporter (VGLUT)–positive neurons in the *Ciona* larva are sensory (i.e., there are no glutamatergic interneurons or efferents). The list of glutamatergic neurons includes the photoreceptors, the gravity-sensitive antenna cells, and the peripheral epidermal sensory neurons (ESNs; Horie et al., 2008a; Kourakis et al., 2019). TH expression is limited to the hypothalamus-like coronet cells (Moret et al., 2005; Lemaire et al., 2021). Other interneurons of the CNS are either VGAT^+^ or VAChT^+^, expressing none of the markers of small-molecule neurotransmitters, and may be peptidergic (Hamada et al., 2011; Kourakis et al., 2019). In the *Ciona* midbrain, VAChT and VGAT are expressed in distinct, nonintermingled domains, with the VAChT domain anterior to the VGAT domain, while in the hindbrain VAChT expression dominates with VGAT expression limited to six ascending motor ganglion interneurons (AMGs) in the dorsal hindbrain and four more caudally positioned ascending contralateral inhibitory neurons (ACINs; Kourakis et al., 2019).

To better understand the role of the glutamate system in *Ciona* larvae and to complement predictions of neurotransmitter use, the expression patterns of the glutamatergic ionotropic receptors (AMPA, NMDA, and kainate) and metabotropic receptors (mGluR) are characterized by in situ hybridization in the present study. While *Ciona* and related animals (the tunicates) are the closest extant relatives of the vertebrates, they have diverged in a number of important ways from the vertebrates. Significantly, the tunicates split from the vertebrates before two whole-genome duplications occurred in the vertebrate lineage (Dehal and Boore, 2005). As a result, tunicates have smaller genomes when compared with vertebrates and in most cases have fewer members of gene families. This relationship is evident in the glutamate receptors: the *Ciona* genome encodes single copies of the AMPA and kainate receptors, as well as single copies of each NMDA receptor subunit (Okamura et al., 2005), and three genes putatively encoding mGlu receptors (Kamesh et al., 2008). Thus, the relative genomic simplicity of *Ciona* greatly simplifies the task of generating a comprehensive view of the expression of the glutamate receptors.

## Material and Methods

### Animals

Adult *Ciona robusta* (also known as *Ciona intestinalis* type A) were collected at the Santa Barbara Harbor. *Ciona* are hermaphrodites. Gametes were dissected from adults and crossed in vitro to generate larvae. All embryos and larvae were cultured at 18°C.

### In situ hybridization and image collection

Whole-mount fluorescent in situ hybridization of larval *C. robusta* was performed using the hybridization chain reaction (HCR) method (v. 3.0, Molecular Instruments; Choi et al., 2018). Complementary RNA probe sets were designed to coding regions for the following *Ciona* genes (unique gene identifiers provided in parentheses): AMPA receptor (XM_018817034.1), NMDA receptor (XM_018816819.1), kainate receptor (XM_026833998.1), metabotropic glutamate receptor 123 (XM_009859697.3), metabotropic glutamate receptor 478 (XM_018816381.1), VGAT (NM_001032573.1), and VAChT (NM_001032789.1). Larvae for in situ hybridization were dechorionated at the midtailbud stage using sodium thioglycolate/protease E or 0.1% trypsin so that left–right asymmetric properties of the CNS would not be disrupted. Briefly, 0.1 g of Na thioglycolate is mixed with 1 ml 0.5% protease E (Sigma-Aldrich P5147) and 320 µl 1 M NaOH in 12 ml seawater. Unhatched tailbud-stage embryos are added to this solution in a 60 mm petri dish whose surface has been coated in 1% agarose/seawater. The embryos are lightly agitated and swirled in the dish until their outer, surrounding vitelline membrane ruptures, allowing the embryos to float free. Embryos are individually transferred to a first wash of seawater in a separate agarose-coated petri dish, after which embryos are batch-transferred through at least four more seawater washes and allowed to develop until reaching the larval stage, when they are fixed for in situ hybridization. Untreated sibling embryos whose chorionic membrane/vitelline envelopes are left intact serve as controls and aid in the staging of the treated embryos. Labeled animals were imaged on an Olympus Fluoview 1000 confocal microscope; postimage analysis used Imaris v6.4.0.0 or Imaris Viewer v9.5.1 as well as Fiji (ImageJ) v2.0.0-rc-69/1.52p.

### Mapping of in situ hybridization patterns to the connectome

The TIFF image stack for in situ HCR expression was converted into voxels through a custom MATLAB and C# script. In MATLAB, the expression value of a given pixel in a *z*-plane of the TIFF stack was converted into an eight-bit integer (0–255). Each *z*-plane in the stack was thus represented by a comma-separated value (CSV) table where the row and column coordinates of the integer expression value are the *x*–*y* coordinates of the corresponding pixel in that plane. Then, with the C# script, each voxel was individually loaded into a custom Unity project for each TIFF stack, where the volume of a voxel was determined by the scale of the pixel and the distance between the *z*-planes. As the voxels were loaded, their meshes were merged and saved as a Unity asset. The final mesh was a 3D construct object of the in situ HCR expression result. The cell shape reconstruction from the connectome data (Ryan et al., 2016) was loaded into the same scene as expression objects for alignment. In order to load the cell shapes, the original data were loaded into the program Reconstruct, where it was exported as a scene. The scene was loaded into Blender where each neuron was exported as a .DXF file for loading into Unity. This consisted of two datasets: a “low resolution” that contained all the data for the brain vesicle (forebrain and midbrain) and a “high resolution” that contained all the data for the motor ganglion (hindbrain). Since VGAT expression is well characterized in both the brain vesicle and the motor ganglion (Kourakis et al., 2019), the expression objects were rotated and overlaid with the cell shapes manually using the following criteria in relation to VGAT expression: in the brain vesicle, the dorsal cap marks the eminens cells, and the two patches, a smaller posterior one and a larger anterior one, on the right side of the brain mark the two photoreceptor groups, PR-I (only pr-9 and pr-10) and PR-II, respectively; in the motor ganglion, the dorsal patch of VGAT marks the AMG (all except AMG-5). After this alignment was done, expression objects with no consistent landmarks, such as VAChT, were brought into view. Using common structures and overlaps across different in situs, all the expression objects were overlapped in reference to each other.

### Analysis of mapped expression patterns

To align coordinates used in mapping, a Unity program using a custom C# script first loaded the expression data voxel by voxel. The program checks if a voxel collides with the mesh of a cell shape. If there was a collision, the expression volume of that voxel was assigned to that neuron. The total expression percent was then determined by taking the total assigned expression volume and dividing it by the total cell volume. The total expression percent of the neuron was then normalized to relative expression by dividing the value by the highest total expressing neuron for that specific in situ. The neuron was predicted to be positive for a particular transcript if the total expression percent was at least 5.5%. The threshold value was determined by first analyzing the hindbrain (also known as the motor ganglion), as the expression of VAChT, VGAT, and AMPAR has been described before (Kourakis et al., 2019, 2021). In particular, VAChT is expressed in known hindbrain neurons ascending motor ganglion-5 (AMG5), the six motor ganglion interneurons (MGINs), and all 10 motor neurons (MNs), while VGAT is expressed in AMG1, 2, 3, 4, 6, and 7. Finally, AMPAR is expressed in the left MNs. Using hindbrain expression values guides, the analysis was performed with a variety of parameters until determining a threshold that aligns with this “ground truth.” These values were used to then determine fore- and midbrain expression.

### Behavioral assays

All larvae were between 25 and 28 h postfertilization (hpf; 18°C). Larval swimming behaviors were recorded in seawater using 10 cm agarose-coated petri dishes to reduce sticking. Image series were collected using a Hamamatsu ORCA-ER camera fitted on a Navitar 7000 macro zoom lens. Two programmable LED lamps (Mightex) were used for the behavioral assays: the 700 nm lamp was used to illuminate the larvae for image capture, and the 505 nm lamp mounted above the Petri dishes was used as the light stimulus for dimming response assays. The dim response movies were recorded at 10 frames per second (fps). The larvae were recorded for 10 s at the initial intensity (3 mW/cm^2^) that was then dimmed (0.3 mW/cm^2^) while image capture continued for 1 min. Larvae were allowed to recover for 5 min before being assessed again. All light intensity readings were taken with an Extech Instruments light meter. For negative phototaxis assays, larvae were recorded at 1 frame per minute for 2 h using constant directional illumination with the 505 nm LED. For pharmacological experiments, MK801 (Tocris Bioscience) was dissolved in seawater to a concentration of 500 μM, and the larvae were exposed to the drug for 10 min before being assessed.

### Code accessibility

The code described in the paper is freely available online at https://github.com/CionaLab/agr and archived at https://doi.org/10.5281/zenodo.12705827. The reconstructed neuron meshes are archived at https://zenodo.org/doi/10.5281/zenodo.12705626. The analysis was performed on a Windows 10 PC.

## Results

### Ionotropic glutamate receptors are expressed in broad, partially overlapping domains

**Figure 2.**
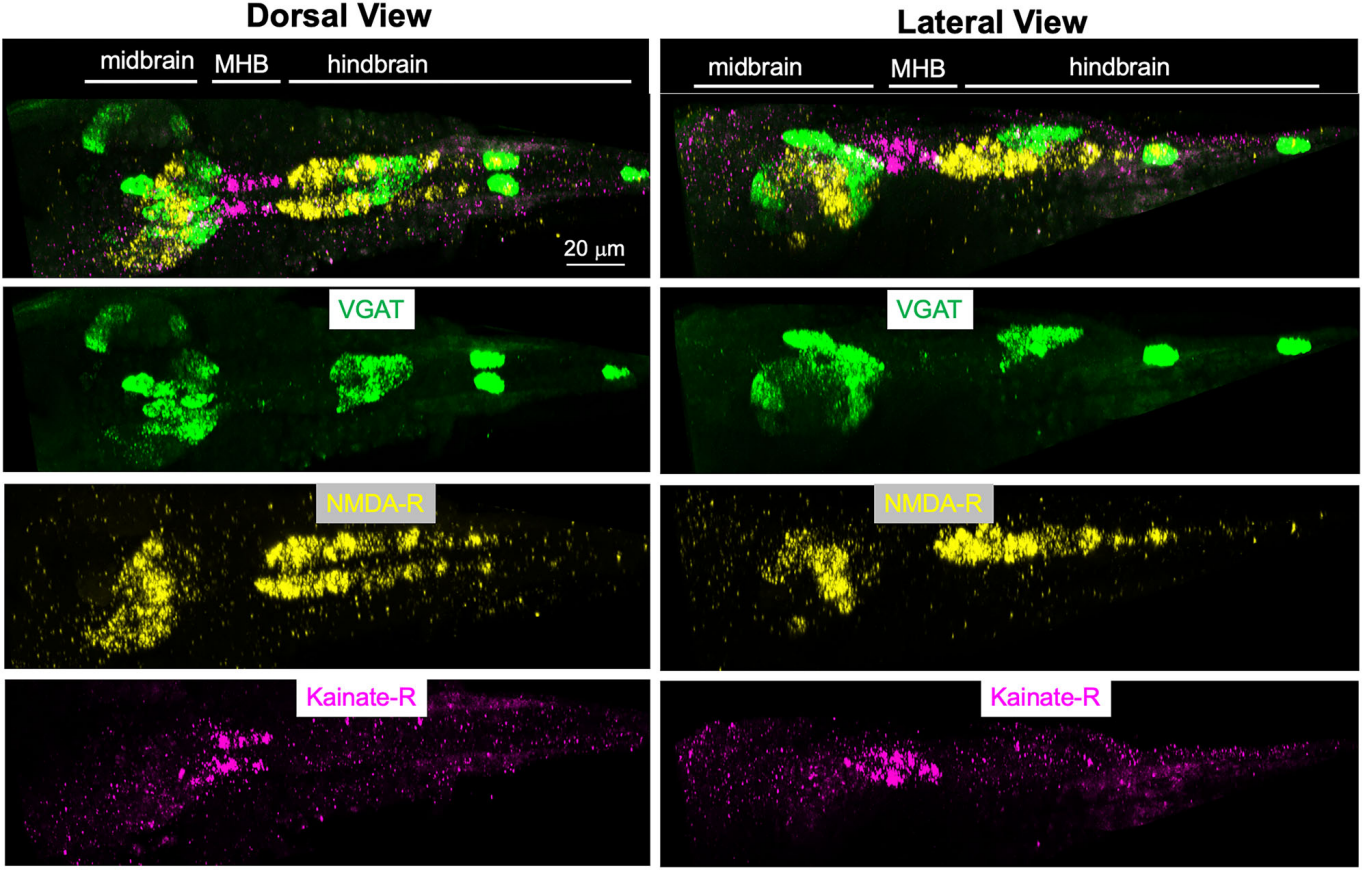
In situ hybridization of *C. robusta* larva for VGAT, NMDAR, and kainate-R. The top panels show the dorsal and lateral composite views of the three expression patterns. The three bottom right and left panels show the individual images that comprise the composite. All images are *z*-projection images from confocal stacks. Anterior is to the left for all panels. MHB, midbrain–hindbrain boundary.

In situ hybridization using the hybridization chain reaction (HCR) method allows for the easy three-dimensional visualization of multiple fluorescently labeled probes in a single sample (Choi et al., 2018). In the present study, probes for ionotropic glutamate receptors (AMPA, NMDA, and kainate), as well as for the cholinergic marker VAChT, and the GABA/glycinergic marker VGAT were tested in groups of three. For the NMDA receptor (NMDAR), the *Ciona* homolog of the GluN1 subunit was used, as it is common to all NMDAR complexes (Paoletti et al., 2013). Figure 1*b* shows the maximum-intensity *z*-projections of confocal images for the expression of VAChT with the AMPA and NMDA receptors in a representative 25 (hpf) larva, while Figure 2 shows the expression of VGAT with NMDA and kainate receptors in an identically staged larva (Videos 1, 2). As seen in Figure 1*b*, the AMPA receptor (AMPAR) expression domain in the fore- and midbrains is more extensive than the NMDA receptor. Expression of AMPAR in the absence of NMDAR was observed in the forebrain (white arrowhead), as well as the posterior midbrain (red arrowhead). We also observed a domain in the midbrain expressing NMDAR, but not AMPAR (Fig. 1*b*, green arrows, lateral view). No expression of either NMDAR or AMPAR was observed in the MHB region.

In contrast to the fore- and midbrains, the expression of NMDAR in the hindbrain was more extensive than that of the AMPAR. With the exception of the seven dorsally located AMG neurons (Ryan et al., 2018), the *Ciona* hindbrain is divided into distinct left and right sides. This is most conspicuous in the five cholinergic motor neurons found on each side, left and right, which innervate tail muscles on the corresponding side (Ryan et al., 2016). However, this left/right symmetry is also present in the interneurons of the ventral hindbrain. Despite the symmetry of the ventral hindbrain at the level of neurons, it has been reported that AMPAR is expressed only on the left side [(Kourakis et al., 2021); our current results are consistent with this distribution (Fig. 1*b*, cyan arrowhead)]. By contrast, we report here that the NMDAR is expressed symmetrically on the left and right sides of the ventral hindbrain (orange arrowhead). Finally, the expression of the kainate receptor was very limited in the CNS and appears to be restricted to a group of neurons in the posterior midbrain (Fig. 2).

### Mapping of expressing neurons to the connectome

**Figure 3.**
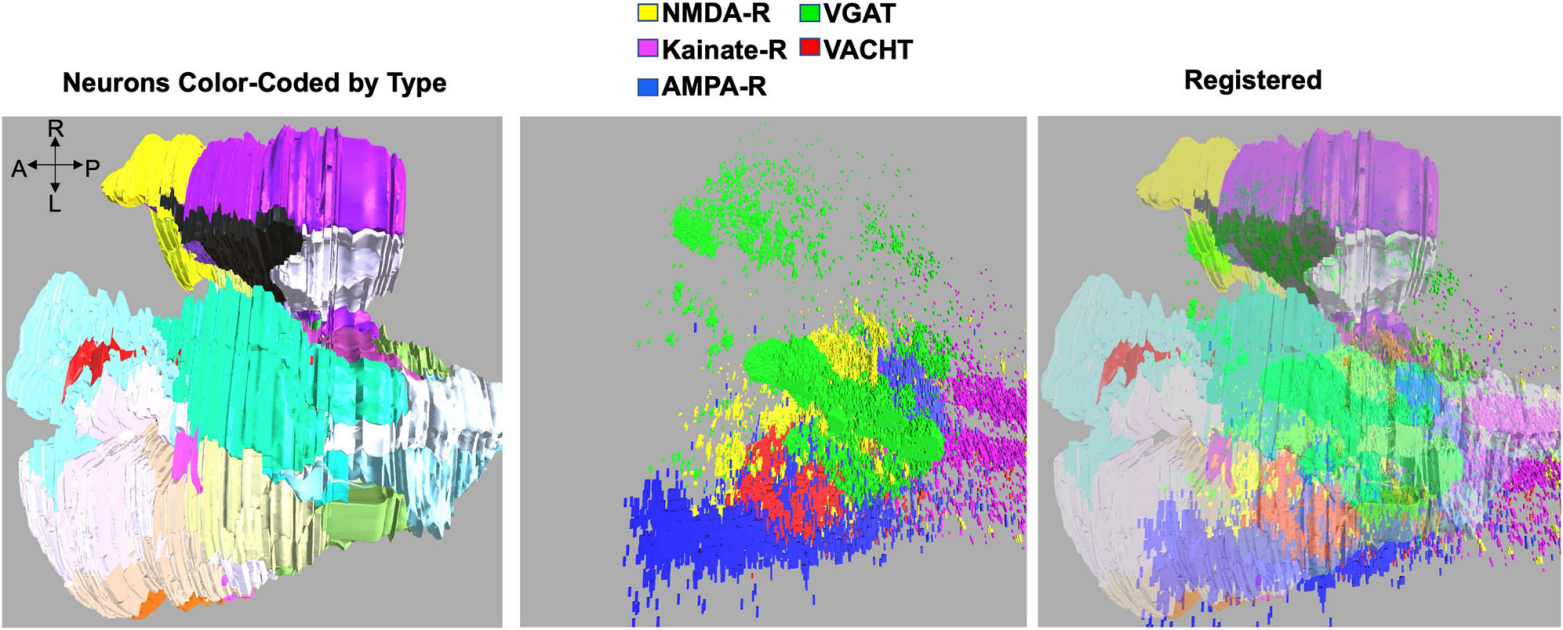
Three-dimensional registration of in situ hybridization data to neurons from the *Ciona* connectome. The left panel shows the fore- and midbrains color-coded by type, as described previously (Ryan and Meinertzhagen, 2019). The center panel shows a composite of fluorescent in situ hybridization signals. The right panel shows the resulting registrations. For the axis: R is right; L is left; A is anterior; P is posterior.

Previous studies have taken advantage of the small number of neurons in the *Ciona* nervous system and the stereotyped cellular anatomy to register in situ expression patterns to the centroids of individual neurons of the *Ciona* connectome in three dimensions, allowing predictions to be made of gene expression at the individual neuron level (Kourakis et al., 2019). Here, in a refinement of this approach, neuron cell volumes from the connectome were used in the registration, rather than centroids. Briefly, three-dimensional in situ image stacks were rendered into three-dimensional expression objects with a custom Unity code and then loaded directly into a custom Unity registration program. In the analysis, we used three VGAT/VAChT/NMDAR, three NMDAR/kainate-R/VGAT, two NMDAR/AMPAR/VAChT, and two AMPAR/VAChT independently derived image stacks. Also loaded were the reconstructed neuron boundaries from the connectome. The in situ expression objects were first manually overlayed on the connectome cell volumes using known neurons in the in situ datasets as anchors (Fig. 3). Once the anchors were aligned, the custom Unity project was run to detect the in situ objects with the connectome neurons.

**Figure 4.**
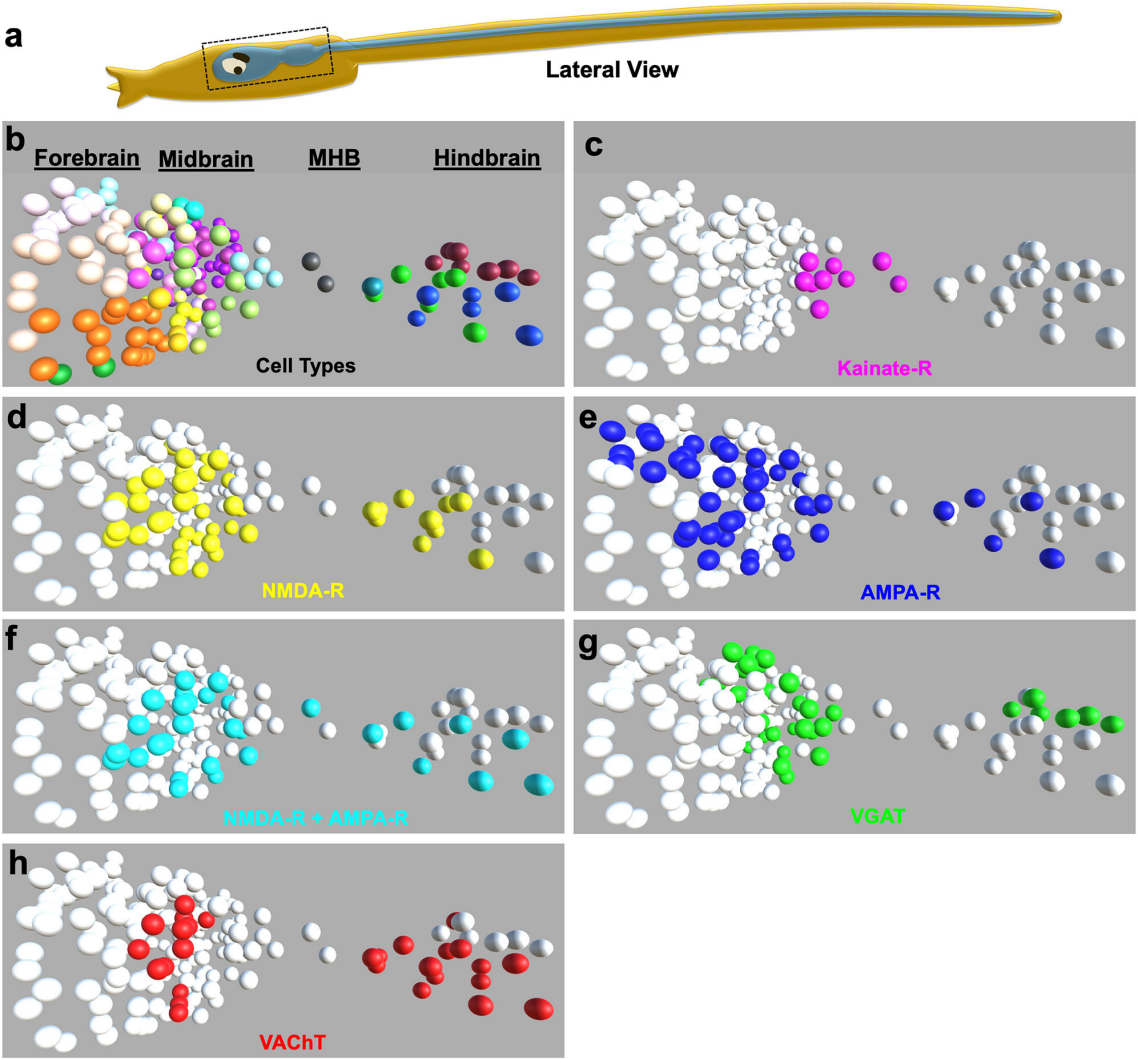
Summary of glutamatergic receptor expression predictions in *Ciona* neurons. ***a***, Lateral view of a *Ciona* larva indicating the brain regions analyzed for glutamate receptor expression in panels ***a–f***. ***b***, The neuron centroids of the forebrain, midbrain, midbrain/hindbrain boundary (MHB) and hindbrain as given by Ryan et al. (2016) and colored by neuron class according to Ryan and Meinertzhagen (2019). See Extended Data Figure 4-1 for key to colors. ***c–e***, Predicted neuronal distribution of kainate, NMDA, and AMPA receptors at larval stage, respectively. ***f***, Predicted neuron coexpressing NMDA and AMPA receptors. ***g***, Predicted expression of VGAT. ***h***, Predicted expression of VAChT. Extended Data Figure 4-1. Key for color-coding of neuron classes is shown in Figure 4*b*. Extended Data Figure 4-2. Dorsal views of Figure 4, panels ***b–h***.

The Unity program was run for the ionotropic glutamate receptors, as well as for VGAT and VAChT to generate expression predictions. The results are summarized in Table 1 and displayed as three-dimensional overlays on the neuron centroids in Figure 4. Figure 4 shows a lateral view of the CNS, which is restricted to the forebrain, midbrain, midbrain/hindbrain junction, and hindbrain (i.e., the spinal cord is not included). The diagram in Figure 4*a* indicates the CNS regions that were analyzed for expression and depicted in the centroid panels (Fig. 4*b–h*). Figure 4*b* shows all neurons identified in these brain regions, which are color-coded as presented in the connectome report (Extended Data Fig. 4-1; Ryan and Meinertzhagen, 2019). Dorsal views of the same expression data are presented in Extended Data Figure 4-2 and highlight the asymmetric left/right expression observed for AMPAR, but not for NMDAR.

10.1523/ENEURO.0306-24.2024.f4-1 Figure 4-1 Key for color-coding of neuron classes shown in Figure 4B. Download Figure 4-1, TIF file.

10.1523/ENEURO.0306-24.2024.f4-2 Figure 4-2 Dorsal Views of Figure 4, panels B-H. Download Figure 4-2, TIF file.

**Table 1.** Predicted expression of VGAT, VAChT, AMPAR, NMDAR, and kainate-R in neurons of the *Ciona* larva as given by the connectome (Ryan et al., 2016)

Cell ID	Cell type	VGAT^+^	VAChT	NMDAR^+^	AMPAR^+^	Kainate-R^+^
95	aaIN	0	+	+	0	0
102	aaIN	0	0	+	0	0
115	aaIN	0	0	+	0	0
107	Ambiguous cells	0	0	0	0	0
177	Ambiguous cells	0	0	0	0	0
Ant1	Antenna	0	0	0	0	0
Ant2	Antenna	0	0	0	0	0
134	AntRN	0	+	+	+	0
135	AntRN	0	+	+	+	0
142	AntRN	+	0	+	+	0
143	AntRN	0	0	+	0	0
147	AntRN	+	0	+	+	0
152	AntRN	+	0	+	+	+
153	AntRN	+	0	+	+	+
159	AntRN	+	0	+	+	0
161	AntRN	+	0	+	+	+
120	AntRN	0	0	+	0	0
90	Bipolar prIN	0	0	0	0	0
92	Bipolar prIN	0	0	0	0	0
3	BVIN	0	0	0	0	0
13	BVIN	0	0	0	+	0
16	BVIN	0	0	0	0	0
18	BVIN	0	0	0	+	0
21	BVIN	0	0	0	0	0
22	BVIN	0	0	0	0	0
24	BVIN	0	0	0	+	0
33	BVIN	0	0	0	+	0
41	BVIN	0	0	0	0	0
42	BVIN	0	0	0	+	0
43	BVIN	0	0	0	0	0
46	BVIN	0	0	0	0	0
138	BVIN	0	0	0	0	0
1	cor-ass BVIN	0	0	0	0	0
2	cor-ass BVIN	0	0	0	0	0
15	cor-ass BVIN	0	0	0	0	0
17	cor-ass BVIN	0	0	0	0	0
23	cor-ass BVIN	0	0	0	0	0
38	cor-ass BVIN	0	0	0	0	0
48	cor-ass BVIN	0	0	0	+	0
50	cor-ass BVIN	0	0	0	0	0
55	cor-ass BVIN	0	0	0	+	0
59	cor-ass BVIN	0	0	0	+	0
60	cor-ass BVIN	0	0	0	+	0
62	cor-ass BVIN	0	0	0	0	0
68	cor-ass BVIN	0	0	+	0	0
70	cor-ass BVIN	0	0	0	0	0
73	cor-ass BVIN	0	0	0	+	0
78	cor-ass BVIN	0	0	0	0	0
79	cor-ass BVIN	0	0	+	0	0
coronet1	Coronet	0	0	0	0	0
coronet 10	Coronet	0	0	+	+	0
coronet11	Coronet	0	0	+	+	0
coronet12	Coronet	0	0	0	0	0
coronet13	Coronet	0	0	0	0	0
coronet14	Coronet	0	0	0	0	0
coronet15	Coronet	0	0	0	0	0
coronet16	Coronet	0	0	0	0	0
coronet2	Coronet	0	0	0	+	0
coronet3	Coronet	0	0	0	0	0
coronet4	Coronet	0	0	0	+	0
coronet5	Coronet	0	0	0	0	0
coronet6	Coronet	0	0	0	+	0
coronet7	Coronet	0	0	0	0	0
coronet8	Coronet	0	0	0	0	0
coronet9	Coronet	0	0	+	+	0
109	Eminens	+	0	0	0	0
99	Eminens	+	0	0	0	0
103	Nonsensory RN	+	0	0	0	0
106	Nonsensory RN	0	0	0	+	0
122	Nonsensory RN	+	0	0	0	0
125	Nonsensory RN	0	+	+	+	0
93	Nonsensory RN	0	0	0	+	0
160	PBV PNIN	+	0	0	0	+
162	PBV PNIN	+	0	0	+	+
163	PBV PNIN	+	0	0	0	+
164	PBV PNIN	0	0	0	0	+
20	PNIN	0	0	0	0	0
25	PNIN	0	0	0	0	0
29	PNIN	0	0	0	0	0
30	PNIN	0	0	0	0	0
4	PNIN	0	0	0	0	0
6	PNIN	0	0	0	0	0
85	PNIN	+	0	0	0	0
61	PNIN	+	0	0	0	0
65	PNIN	+	0	0	0	0
88	PNIN	+	0	0	0	0
131	PN RN	+	0	0	0	0
prIII-101	PR (III)	0	0	0	0	0
prIII-110	PR (III)	0	0	0	0	0
prIII-113	PR (III)	0	0	0	0	0
prIII-114	PR (III)	0	0	0	0	0
prIII-6	PR (III)	0	0	0	0	0
prIII-7	PR (III)	0	0	0	0	0
prIII-84	PR (III)	0	0	0	0	0
108	pr-AMG RN	0	0	0	0	0
116	pr-AMG RN	+	0	+	0	0
124	pr-AMG RN	+	0	0	0	0
127	pr-AMG RN	0	0	+	0	0
140	pr-AMG RN	0	+	+	+	0
157	pr-AMG RN	+	0	+	+	0
74	pr-AMG RN	0	0	0	0	0
94	pr-AMG RN	+	0	+	0	0
123	pr-BTN RN	+	0	+	0	0
130	pr-BTN RN	+	0	+	0	0
105	pr-cor RN	0	+	+	+	0
112	pr-cor RN	0	+	+	+	0
119	pr-cor RN	0	+	+	+	0
100	prRN	0	+	+	+	0
121	prRN	0	+	+	+	0
126	prRN	0	+	+	+	0
80	prRN	0	0	0	0	0
86	prRN	0	+	+	+	0
96	prRN	0	+	+	+	0
AMG1	AMG	+	0	0	0	0
AMG2	AMG	+	0	0	0	0
AMG3	AMG	+	0	0	0	0
AMG4	AMG	+	0	0	0	0
AMG5	AMG	0	+	0	0	0
AMG6	AMG	0	0	0	0	0
AMG7	AMG	0	0	0	0	0
ddNL	ddN	0	+	+	+	0
ddNR	ddN	0	+	+	0	0
MGIN1L	MGIN	0	+	+	+	0
MGIN1R	MGIN	0	+	+	0	0
MGIN2L	MGIN	0	+	+	+	0
MGIN2R	MGIN	0	+	+	0	0
MGIN3L	MGIN	0	+	+	+	0
MGIN3R	MGIN	0	+	+	0	0
MN1L	MN	0	+	+	+	0
MN1R	MN	0	+	+	0	0
MN2L	MN	0	+	0	+	0
MN2R	MN	0	+	0	0	0
MN3L	MN	0	+	+	+	0
MN3R	MN	0	+	+	0	0
165	Neck	0	+	+	+	+
166	Neck	0	+	+	0	+
pr1	PR (I)	0	0	0	0	0
pr10	PR (I)	+	0	0	0	0
pr11	PR (I)	0	0	0	0	0
pr12	PR (I)	0	0	0	0	0
pr13	PR (I)	0	0	0	0	0
pr14	PR (I)	0	0	0	0	0
pr15	PR (I)	0	0	0	0	0
pr16	PR (I)	0	0	0	0	0
pr17	PR (I)	0	0	0	0	0
pr18	PR (I)	0	0	0	0	0
pr19	PR (I)	0	0	0	0	0
pr2	PR (I)	0	0	0	0	0
pr20	PR (I)	0	0	0	0	0
pr21	PR (I)	0	0	0	0	0
pr22	PR (I)	0	0	0	0	0
pr23	PR (I)	0	0	0	0	0
pr3	PR (I)	0	0	0	0	0
pr4	PR (I)	0	0	0	0	0
pr5	PR (I)	0	0	0	0	0
pr6	PR (I)	0	0	0	0	0
pr7	PR (I)	0	0	0	0	0
pr8	PR (I)	0	0	0	0	0
pr9	PR (I)	0	0	0	0	0
pr-a	PR (II)	+	0	0	0	0
pr-b	PR (II)	+	0	0	0	0
pr-c	PR (II)	+	0	0	0	0
pr-d	PR (II)	+	0	0	0	0
pr-e	PR (II)	+	0	0	0	0
pr-f	PR (II)	+	0	0	0	0
pr-g	PR (II)	+	0	0	0	0

### Expression of mGlu receptors in the peripheral nervous system

**Figure 5.**
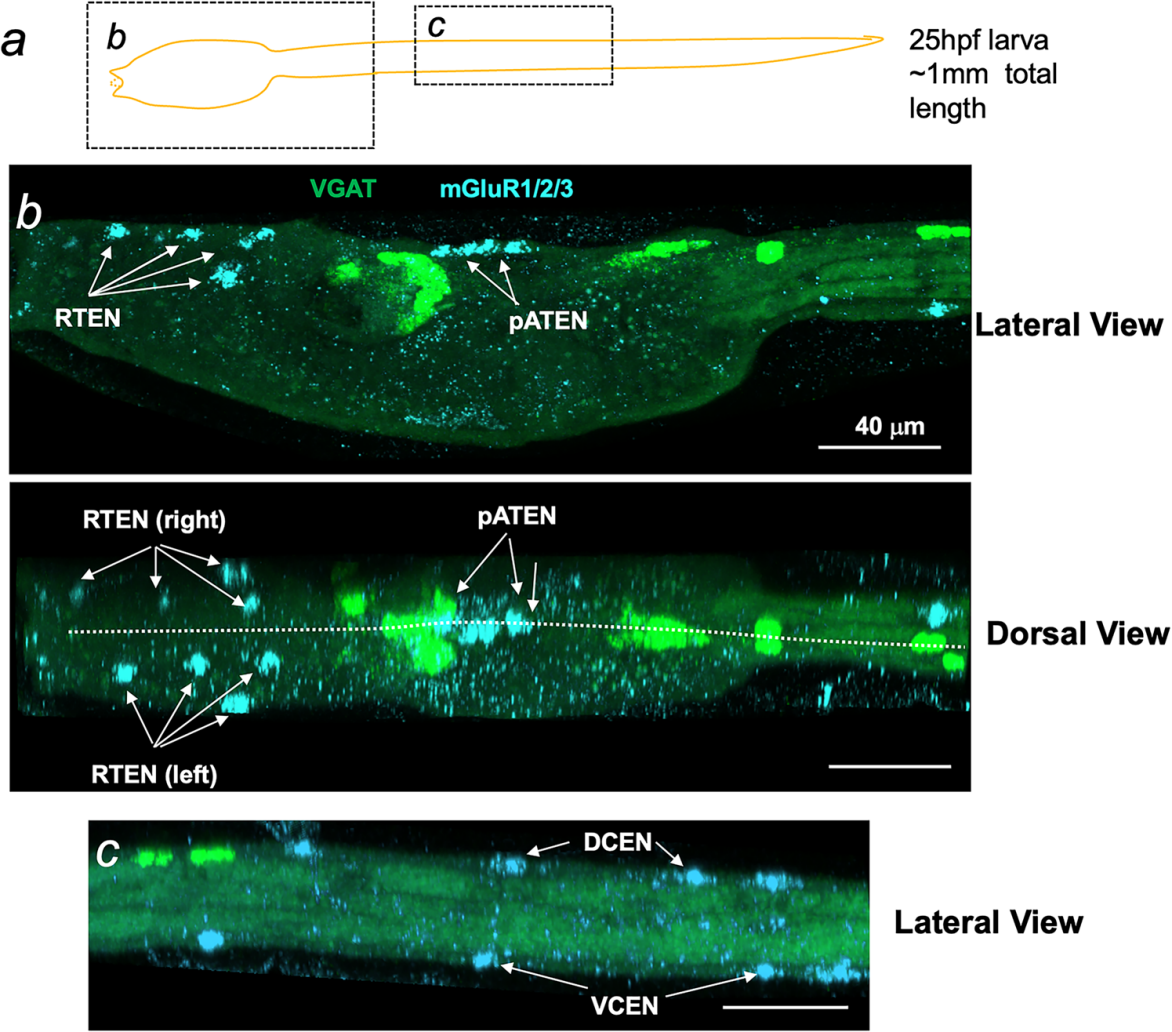
Expression of mGluR1/2/3 by in situ hybridization. ***a***, A diagram of *Ciona* larva indicating regions shown in panels ***b*** and ***c***. ***b***, mGluR1/2/3 expression in the trunk of a *Ciona* larva. The arrows point to mGluR1/2/3-expressing epidermal sensory neurons. RTEN, rostral trunk epidermal neurons; aATEN, anterior apical trunk epidermal neurons; pATEN, posterior apical trunk epidermal neurons. Both dorsal and lateral views are shown. The dotted line in the dorsal view indicates the midline. ***c***, mGluR1/2/3 expression in the tail of a *Ciona* larva. DCEN, dorsal caudal epidermal neurons; VCEN, ventral caudal epidermal neurons. Extended Data Figure 5-1. Expression of mGlu receptors (mGluR) in the *Ciona* larvae single-cell RNaseq dataset from Cao et al. (2019). The top left panel shows cells clustered by UMAP analysis and color-coded by tissue type. The remaining three panels show the distribution of the three putative mGlu receptors in the clusters. Notice that mGluR1/2/3 is more highly expressed than the other two.

Analysis of the *Ciona* genome revealed the presence of three putative metabotropic glutamate receptors (mGluR; Kamesh et al., 2008). Based on their orthologies to vertebrate mGluRs, they were named mGluR1/2/3, mGluR4/7/8, and mGluR1/4/7. Our analysis of a previously published single-cell RNAseq (scRNAseq) dataset (Cao et al., 2019) indicated that mGluR1/2/3 was expressed at higher levels at the larval stage than the other two mGluRs (Extended Data Fig. 5-1). In situ hybridization for mGluR1/2/3 revealed expression in the ESNs, but no apparent central nervous system expression (Fig. 5). This observation agrees with the distribution of mGluR1/2/3 transcripts among the tissue types for the scRNAseq dataset. While mGluR1/2/3 expression was widespread among the ESNs, including the rostral trunk epidermal neurons (RTEN), the posterior apical trunk epidermal neurons (pATEN), and the dorsal and ventral caudal epidermal neurons (DCEN and VCEN, respectively), we did not observe expression in the anterior rostral trunk epidermal neurons, which are found between the RTENs and the pATENs [Fig. 5; for a complete description of *Ciona* epidermal sensory neurons, refer to Ryan et al. (2018)]. While the 3D coordinates for the ESNs were not included in the connectome dataset, the ESNs are well described and easily identified, allowing in situ expression patterns to be confidently attributed. An in situ hybridization was also performed for mGluR4/7/8, and no expression was detected—consistent with the low expression indicated by the scRNAseq dataset. The scRNAseq dataset indicates that mGluR1/4/7 is also expressed at a lower level than mGluR1/2/3, and no in situ hybridization was attempted. However, the tissue distribution of mGluR1/4/7 is similar to that of mGluR4/7/8, with expression concentrated in the epidermis (Extended Data Fig. 5-1).

10.1523/ENEURO.0306-24.2024.f5-1 Figure 5-1 Expression of mGlu receptors (mGluR) in the Ciona larvae single cell RNaseq dataset from (Cao et al., 2019) . The top left panel shows cells clustered by UMAP analysis and color-coded by tissue type. The remaining three panels show the distribution of the three putative mGlu receptors in the clusters. Notice that mGluR123 is more highly expressed than the other two. Download Figure 5-1, TIF file.

### NMDA receptors are required for sensorimotor responses

*Ciona* larvae display two distinct visuomotor behaviors. In the presence of a constant directional illumination, they display negative phototaxis. In contrast, rapid dimming of light evokes circular swimming—thought to be an evasion mechanism to avoid predation (Salas et al., 2018). Moreover, these two behaviors are mediated by distinct sets of photoreceptors that act through distinct neural circuits (Kourakis et al., 2019). It was previously reported that the AMPAR inhibitor perampanel blocked negative phototaxis, but not the ability of larvae to respond to dimming light (Kourakis et al., 2019). This result is consistent with the predicted differential expression of AMPAR on the primary interneuron targets of the photoreceptors in the midbrain, known as the photoreceptor relay neurons (prRNs) and the photoreceptor-ascending motor ganglion relay neurons (pr-AMG RNs). Specifically, the prRNs are hypothesized to directly mediate phototaxis, while the pr-AMG RNs are hypothesized to mediate the dimming response (Kourakis et al., 2019). Our analysis here predicts that the prRNs express both AMPAR and NMDAR (Table 1). In contrast, a subset of the pr-AMG RNs express NMDAR but not AMPAR (Table 1).

**Figure 6.**
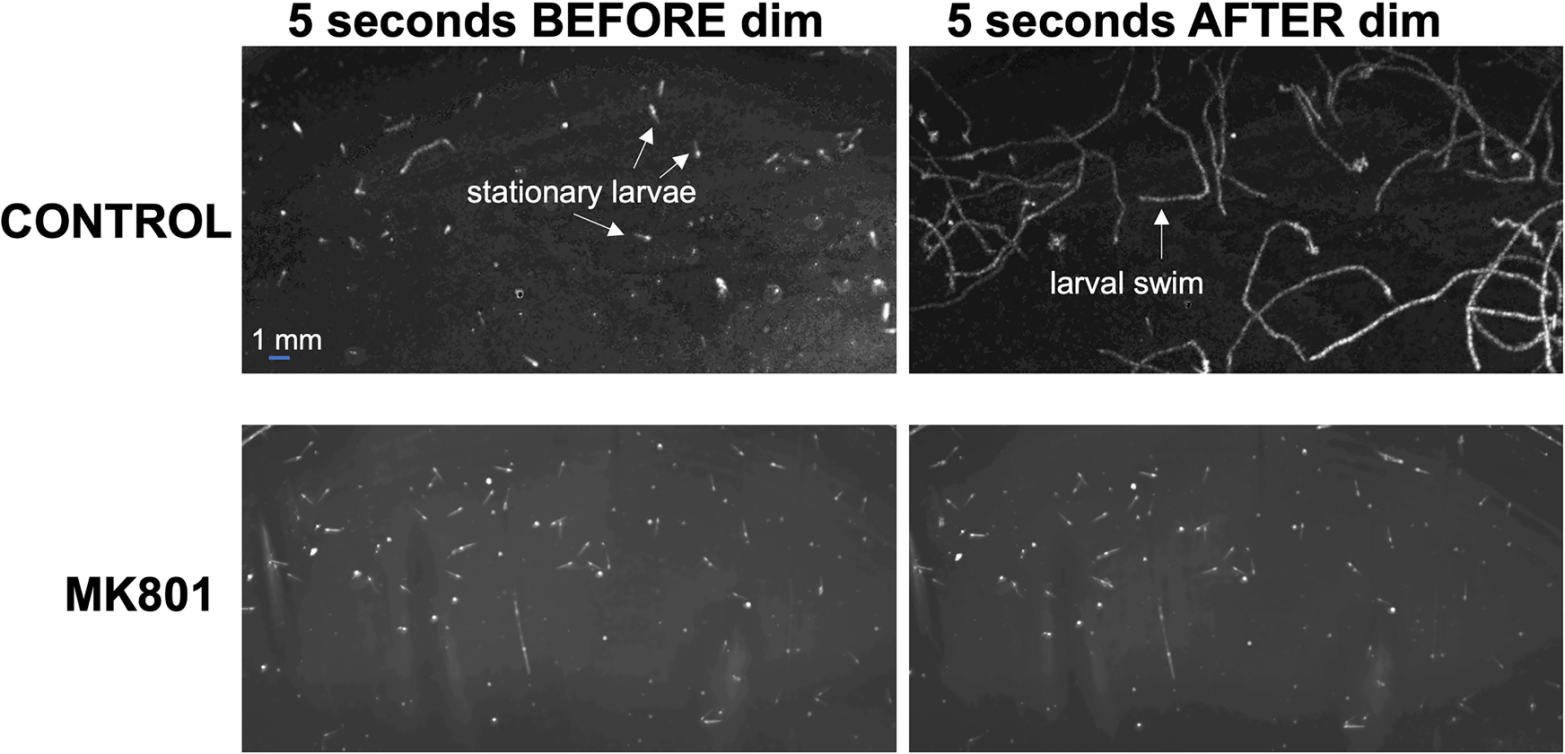
MK801 blocks the dimming response. The left two panels show *Ciona* larvae in a temporal projection of the 5 s preceding light dimming for both control and MK801-treated larvae. The lines represent larval paths within the 5 s projection. Most larvae are stationary in the 5 s preceding the dimming event. In the 5 s following light dimming, the CONTROL larvae are observed swimming (white lines), while the MK801-treated larvae do not respond.

**Figure 7.**
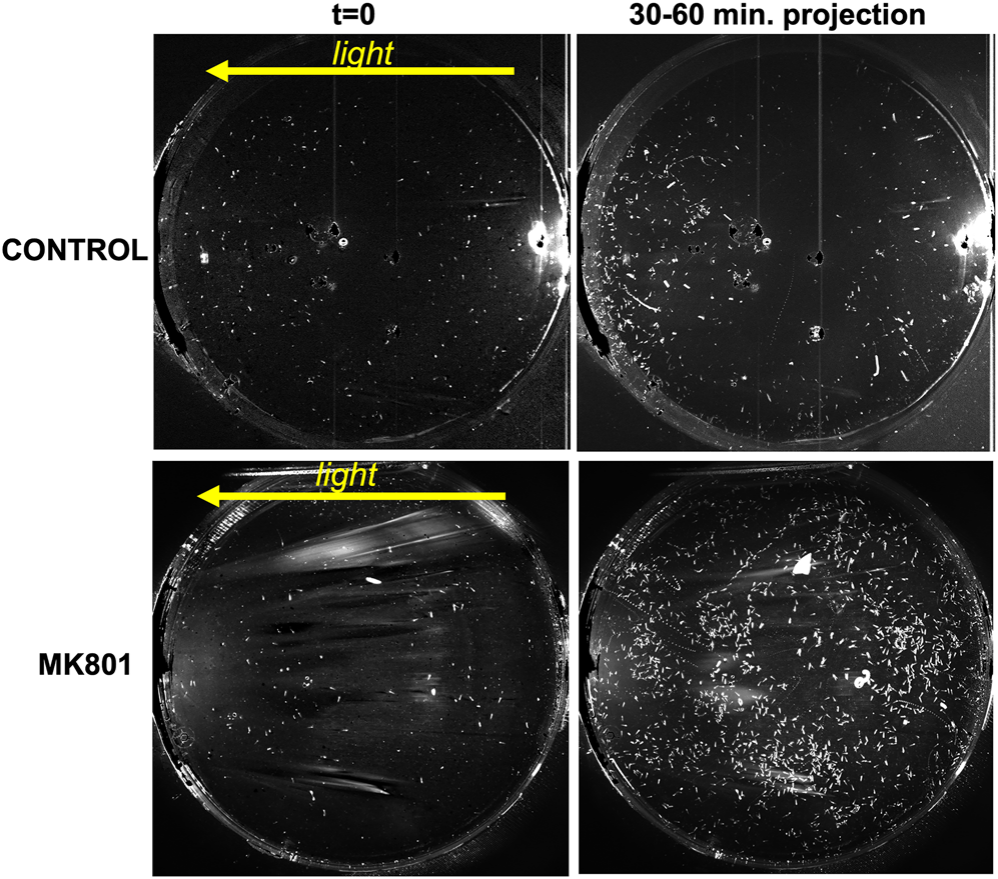
MK801 blocks the phototaxis response. The left two panels show the first frame of the phototaxis assay with the yellow arrow indicating the direction of the light. The right two panels show temporal projects from 30 to 60 min in directional light. *Ciona* larvae can be seen accumulated on the left in the control and more dispersed in the MK801-treated.

In order to assess the role of NMDARs in visuomotor behaviors we used the noncompetitive NMDA receptor antagonist MK801. MK801 shows strong inhibition and specificity of NMDARs in both vertebrates and invertebrates (Wong et al., 1986; Vogeler et al., 2021). The effect of NMDAR inhibition was first tested in a dimming assay (Salas et al., 2018). Larvae were recorded using far-red illumination (700 nm), while a 505 nm LED lamp was dimmed from 3 to 0.3 mW/cm^2^ midway through the recording. We observed that unlike the AMPAR antagonist perampanel, MK801 completely inhibited the dimming response (Fig. 6 and Video 3). Figure 6 shows the temporal projections of the movies for the 5 s immediately before and after the dim. Before the dim, larvae are mostly stationary, but in response to dimming, the control larvae immediately initiate swimming, which is seen as lines in the time-projection image (Fig. 6, labels). In contrast, larvae treated with 0.5 mM MK801 did not respond to dimming (bottom two panels). A similar result was observed in a phototaxis assay (Fig. 7 and Video 4). In the phototaxis assay, larvae are placed in a petri dish with a light source of constant intensity from one side and recorded for 1 h (Salas et al., 2018). At the end of the assay period, phototaxis is evident by the accumulation of larvae at the side of the petri dish furthest from the light source. Figure 7 shows the images from Video 4 of the control and MK801-treated larvae at the start of the phototaxis assay (*t* = 0), in which the larvae can be seen evenly distributed across the Petri dishes (left panels). The right panels show a temporal projection of the movie from time points 30–60 min. The accumulation of larvae at the left side of the petri dish (away from the light) is evident in the control, but not in the MK801-treated sample. However, as is evident in Video 4, the MK801-treated larvae are swimming, indicating that their ability to swim is not impaired, only their ability to perform phototaxis. Thus, the NMDAR antagonist MK801 is effective at inhibiting both the dimming response and phototaxis, unlike the AMPA antagonist perampanel, which only inhibits phototaxis.

## Discussion

The small number of neurons in the *Ciona* larva, together with the published connectome and easily quantified sensorimotor behaviors, make it a powerful model for neuron circuit analysis. Moreover, *Ciona* is currently the only chordate for which a complete analysis of all neurons in a nervous system has been reported. The key to making testable models of neural circuits is a knowledge of the distribution of neurotransmitter receptors. We have presented here a comprehensive prediction of glutamate receptor expression in the *Ciona* larval nervous system at the individual neuron level. Also presented are analyses for the distribution of VGAT^+^ and VAChT^+^ neurons, which expand on a previous report (Kourakis et al., 2019) to additional neurons in the CNS and resolve the neurotransmitter use of neurons that were previously ambiguous. The challenge of mapping gene expression data to the connectome is that the fully annotated connectome exists for only a single individual, while in situ hybridization results can be generated from multiple individuals. The ability to map expression patterns to individual neurons of the connectome with confidence would only be possible if the CNS shows stereotypy. The issue of stereotypy was addressed in a previous publication (Kourakis et al., 2019), which found that the number and spatial distribution of neurons, as determined by in situ hybridization, in the hindbrain was highly stereotyped, while the stereotypy was less strong in the midbrain (the forebrain was unexplored in this study). However, it was found that the non-intermingled expression domains of VAChT and VGAT in the midbrain were invariable. A second limit to the confidence of this approach is the fact that the connectome of only one larva has been determined, and thus, while it has been found, for example, that the number and gene expression patterns of the neurons in the hindbrain are highly stereotyped, the same cannot be assumed for their connectivity. It is also important to note that our results are for a specific developmental stage of the larva (∼25 h postfertilization at 18°C). Changes in *Ciona* larval behavior have been on timescales as short as 2–3 h (Salas et al., 2018; Bostwick et al., 2020), so it is possible that the expression of the glutamate receptors is temporally dynamic within the larva. Nevertheless, in the temporal window that was analyzed here, the distribution of glutamate receptors in the *Ciona* nervous system provides new insight into neural circuits driving sensorimotor behaviors.

### Glutamate receptors in the forebrain

The *Ciona* larval forebrain has been less thoroughly investigated in terms of function and stereotypy than other parts of the CNS. Interestingly, there are no VAChT- or VGLUT-expressing neurons in the forebrain and only two VGAT-expressing neurons (Chung et al., 2023). However, one of the forebrain VGAT neurons (cor-assBVIN78) was reported to be not only highly stereotyped but to play an essential inhibitory role in phototaxis (Chung et al., 2023). However, this neuron is not predicted to express any of the glutamate receptors. Expression of the glutamate receptors is predicted in six of the fourteen forebrain coronet cells, although the connectome does not predict that any of the coronet cells are postsynaptic to glutamatergic neurons (Ryan et al., 2016; Table 1). There are also five brain vesicle interneurons (BVINs) that are predicted to express AMPARs, two of which (BVINs 13 and 42) are predicted to be postsynaptic to the photoreceptors and another two (BVINs 24 and 33) predicted to be postsynaptic to the antenna cells. In addition to the BVINs, four coronet-associated brain vesicle interneurons (cor-assBVINs) are predicted to express AMPAR, although only one, cor-assBVIN 60, is predicted to be postsynaptic to a glutamatergic neuron (Antenna neuron 2).

A conspicuous group of neurons in the forebrain are the photoreceptors. A surprising observation to come of this study is that while most of the *Ciona* larval photoreceptors are glutamatergic, and their targets in the midbrain (prRNs and pr-AMG RNs) express both AMPARs and NMDARs, no evidence was found of expression of any of the glutamate receptors in the photoreceptors themselves despite the connectome predicting extensive chemical synapses between them (Ryan et al., 2016). While a subset of the photoreceptors are GABAergic, suggesting that some of the chemical synapses between photoreceptors could be via GABA receptors, the glutamatergic photoreceptors are also presynaptic to other photoreceptors, suggesting that neurotransmitters other than glutamate or GABA might be involved. The expression of the adrenoceptor ADRa2a in photoreceptors suggests a candidate synaptic pathway for communication between *Ciona* larval photoreceptors (Borba et al., 2021).

### Glutamate receptors in the midbrain

In contrast to the forebrain, expression of AMPAR and NMDAR in the midbrain was much more extensive. The anterior midbrain receives input from the photoreceptors, while the posterior midbrain receives synaptic input from the otolith-associated antenna sensory neurons, which mediate gravitaxis (Ryan et al., 2016; Bostwick et al., 2020). The target of the two VGLUT^+^ antenna neurons, the VGAT^+^ antenna relay neurons (AntRNs), all express either NMDAR, AMPAR, or both (Table 1; Figs. 4, 5). The 11 AntRNs have a surprisingly complex synaptic connectivity, with some receiving input from one antenna neuron, some from the other, and some from both (Ryan et al., 2016). It was previously reported that perampanel was effective at blocking gravitaxis at 21 hpf, but not at 25 hpf, leading us to speculate, based on the presence of extensive gap junctions between the antenna cells and AntRNs, that the synapse matured from chemical to electrical during that temporal window (Bostwick et al., 2020). The results here, which suggest heterogeneity of glutamate receptor expression, only add to the apparent complexity and call for further investigation.

**Figure 8.**
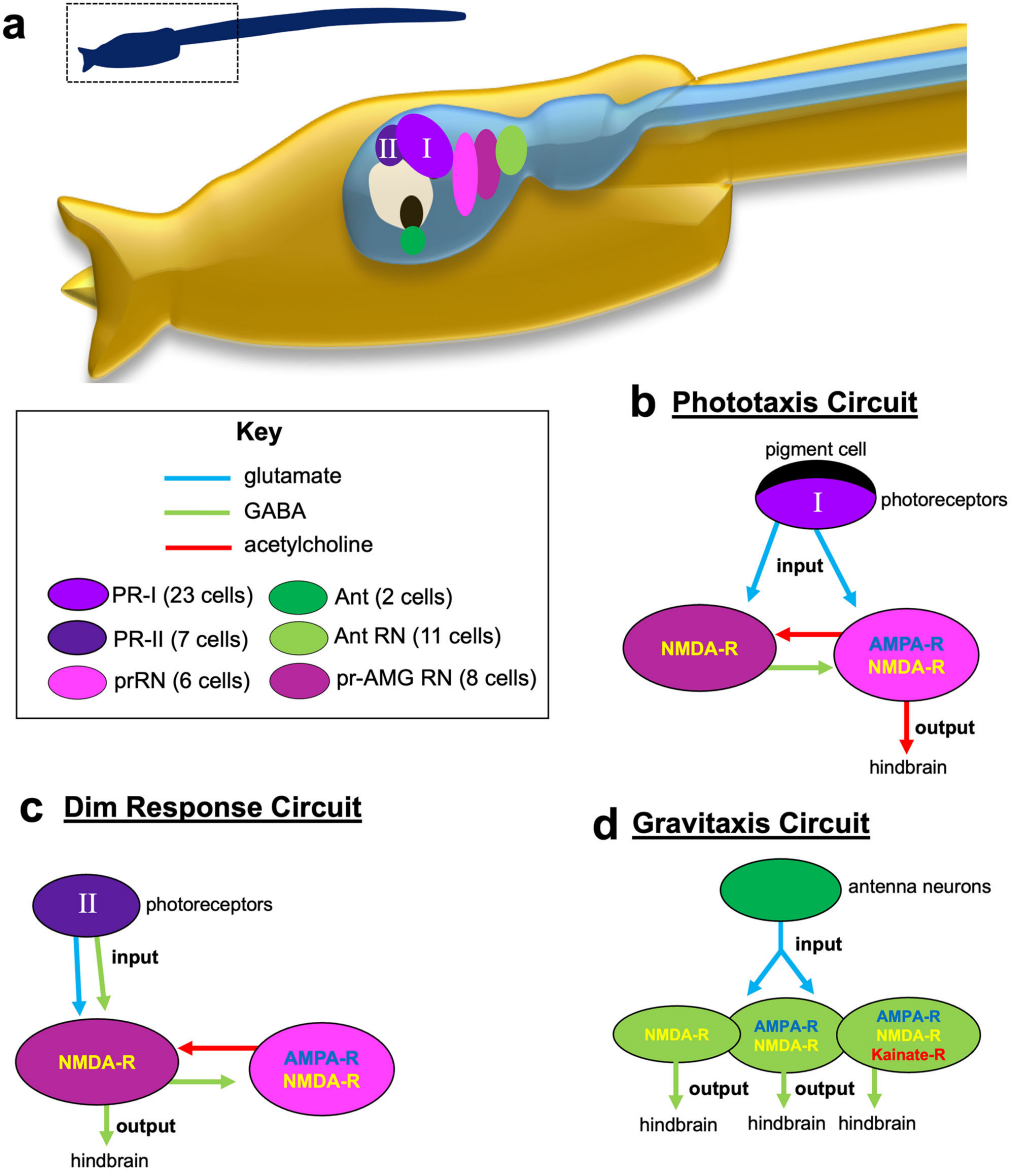
Model neural circuits with contributions by glutamate receptors. ***a***, Overview of central nervous system visual and gravitactic circuits in a larval *Ciona*. The left and right centers in the hindbrain contain secondary interneurons and motor neurons (data not shown). ***b***, Phototaxis circuit. Note that the PR-I photoreceptors (input) project to both the prRNs and the pr-AMG RNs, but that output is exclusively from the prRNs. ***c***, Dim response circuit. This response involves the PR-II photoreceptors, which project exclusively to the pr-AMG RNs. ***d***, Gravitaxis circuit. The gravity-sensitive antenna neurons project to VGAT^+^ primary interneurons that are heterogeneous in their expression of ionotropic glutamate receptors. In all panels, neuron classes are named and color-coded according to Ryan and Meinertzhagen (2019). OT, otolith; PR-I, photoreceptor group I; PR-II, photoreceptor group II; prRN, photoreceptor relay neurons; pr-AMG RN, photoreceptor-ascending MG relay neurons; Ant, antenna neurons; Ant RN, antenna relay neuron.

Photoreceptor input to the *Ciona* larval midbrain is even more complex than antenna cell input. The visual organ of *Ciona*, the ocellus, contains two distinct groups of photoreceptors, PR-I and PR-II (Horie et al., 2008b; Kourakis et al., 2019). PR-I consists of 23 photoreceptors (21 VGLUT^+^, one VGAT^+^, and one VGLUT^+^/VGAT^+^), all of which project their outer segments into the ocellus pigment cell. The direction-dependent shading of the photoreceptors by the pigment cell as larvae perform short orienting swims provides a cue to the direction of light and thereby mediates negative phototaxis (Salas et al., 2018). In contrast, the seven PR-II photoreceptors (three VGAT^+^ and four VGLUT^+^/VGAT^+^) are not associated with the pigment cell and are sensitive to light from all directions and thereby mediate a light-dimming behavior (Salas et al., 2018). The PR-I photoreceptors project axons to two distinct classes of primary interneurons in the midbrain, the prRNs and the pr-AMG RNs (Fig. 8*a*; Ryan et al., 2016). At least five of the six prRNs are predicted to be cholinergic and express both AMPAR and NMDAR (Table 1; Fig. 4). Our model of phototaxis hypothesizes that the cholinergic prRNs relay excitatory input from the VGLUT^+^ photoreceptors to secondary cholinergic interneurons in the hindbrain and then to the motor neurons (Kourakis et al., 2019). However, the other midbrain interneuron class targeted by the PR-I photoreceptors, the pr-AMG RNs, is predicted to be mostly inhibitory. The prediction that PR-I photoreceptors project to both excitatory (prRNs) and inhibitory (pr-AMG RNs) relay neurons initially appears to be difficult to account for within the model. However, our observation here that the pr-AMG RNs express NMDARs, which have a modulatory role, but not AMPARs, provides a possible explanation (Fig. 8). Moreover, a hypothesis was previously put forward that the projection of the PR-I photoreceptors to both excitatory and inhibitory primary interneurons constitute an incoherent feedforward loop circuit motif that functions in visual processing to generate the observed fold-change detection behavior in phototaxis (Borba et al., 2021). In fold-change detection, the response scales with the magnitude of change in sensory input, not with the absolute value of the input (Adler and Alon, 2018). The presence of NMDARs, but not AMPARs, on the pr-AMG RNs fits well with a hypothesized modulatory role in this circuit. Moreover, further investigation on NMDAR subunit composition in the various neurons characterized here may be informative. Finally, transcripts detected by HCR in situ likely tell only part of the story, and investigation of the distribution and quantity of the glutamate receptor proteins by immunolabeling is likely to be informative.

**Movie 1.** Three-dimensional views of multiplex in situ hybridization for VAChT (red), AMPAR (blue), and NMDAR (yellow). [View online]

**Movie 2.** Three-dimensional views of multiplex in situ hybridization for VGAT (green), NMDAR (yellow), and Kainate-R (magenta). [View online]

**Movie 3.** Video of *Ciona* larval dimming response. Both control and MK801-treated larvae are shown. The video was recorded with constant far-red LED illumination (700 nm; which the larva cannot see) and with a red filter on the camera lens. A second LED at 505 nm (visible to the larvae) was illuminated for the first half of the recording, then turned off (indicated by DIM in the video). The video plays in real time. [View online]

**Movie 4.** Video of *Ciona* larval phototaxis behavior. Both control and MK801-treated larvae are shown. The larvae were recorded at 1 frame/minute. The direction of the directional light (505 nm) is indicated. The movie shows 60 min compressed to 10 s (i.e., 360× speed). [View online]

The PR-II photoreceptors project only to the pr-AMG RNs (Fig. 8*b*). All of the PR-II photoreceptors are VGAT^+^ (and thus likely inhibitory), with a subset being dual VGLUT^+^/VGAT^+^. Because their sole synaptic targets, the pr-AMG RNs, also appear to be mostly inhibitory, this led to the hypothesis that the dimming response is mediated by disinhibition, a hypothesis supported by pharmacology (Kourakis et al., 2019). However, the dual release of glutamate and GABA by a subset of the PR-II photoreceptors was not easily accounted for in the disinhibition model. Our finding here that a set of the pr-AMG RNs express NMDAR, but not AMPARs (Table 1, Fig. 4), agrees well with the disinhibition model. In other words, glutamate could serve a modulatory role in the dimming response circuit, perhaps acting on GABA receptors, as has been shown previously (Marsden et al., 2007). Moreover, like the phototaxis behavior, the dimming response shows fold-change detection, although with a different putative circuit motif than in the phototaxis circuit (Borba et al., 2021), and modulation of GABA receptors could serve as the “memory” component of the fold-change detection circuit, as has been described for other systems showing fold-change detection (Lyashenko et al., 2020).

### Glutamate receptors in the hindbrain

AMPAR transcripts have been reported as present asymmetrically in the hindbrain, with expression only observed on the left side (Kourakis et al., 2021). In contrast, we report here that NMDAR transcripts were observed equally in the left and right hindbrain. The presence of glutamate receptors in the hindbrain initially appears to be paradoxical, as none of the hindbrain neurons expressing AMPAR and NMDAR receive direct sensory input (Ryan et al., 2016, 2018), and thus no direct glutamatergic synaptic input. By contrast, the AMG neurons of the dorsal hindbrain are primary synaptic targets of glutamatergic ESNs called the posterior apical trunk epidermal neurons (pATENs; Ryan et al., 2018), yet have no detectable glutamate receptor expression (Table 1). However, the connectome predicts extensive electrical synapses between the peripheral sensory neurons and the AMGs, suggesting the transmission to the AMGs is not chemical. Nevertheless, since the peripheral sensory neurons are the only glutamatergic neurons to enter the hindbrain, we speculate that they may signal to the glutamate receptor–expressing hindbrain neurons extrasynaptically, a phenomenon which has been observed previously for glutamate signaling, with the distance between the peripheral sensory neuron termini and their putative hindbrain targets well within the diffusion range of glutamate (Pál, 2018).

### Kainate-R and mGluR expressions

In addition to investigating the expression of AMPAR and NMDAR, we also examined kainate and metabotropic glutamate receptors. Kainate receptors are ionotropic and appear to have functions both pre- and postsynaptically (Contractor et al., 2011). We observed kainate-R expression in a distinct set of neurons in the posterior midbrain that are predicted to correspond to the posterior BV peripheral interneurons (PBV-PNINs; Table 1 and Fig. 4). Consistent with this identity for the kainate-expressing neurons, the PBV-PNINs are direct targets of the glutamatergic ESNs called the rostral trunk epidermal neurons (RTENs; Horie et al., 2008a; Ryan and Meinertzhagen, 2019). It is not evident why the RTEN targets are unique in their expression of kainate receptors, while the targets of other ESNs, such as the AMGs, appear not to express glutamate receptors.

Of the three predicted metabotropic glutamate receptors (mGluR; Fig. 2), we only found evidence for the expression of one of them and only in peripheral sensory neurons (Fig. 8). The function of this mGluR is not known, but we speculate that it may play a role in signaling between the ESNs to mediate attenuation of the touch response.

### Behavioral requirements for NMDAR and AMPAR

Our observation that treatment of larvae with MK801 blocked visuomotor responses (phototaxis and dim response) came as a surprise. Because of the documented modulatory role of NMDARs, we speculate that tonic activation of NMDAR, perhaps extra synaptically in some cases, may be necessary to maintain sensorimotor responses. Because of the widespread distribution of NMDARs in the CNS, including in motor neurons and MGINs, which are common to all sensorimotor circuits, it is not possible to attribute the result to blocking NMDARs in a particular neuron class. Neuron-specific targeting methods, such as CRISPR, will be required to assess the role of NMDARs in particular circuits. Nevertheless, the observation that inhibition of AMPARs with perampanel blocked only phototaxis, while MK801 blocked both the dimming response and phototaxis, is consistent with the AMPAR negative and NMDAR positive midbrain neurons mediating the dimming response.

## Synthesis

Reviewing Editor: Mark Laubach, American University Decisions are customarily a result of the Reviewing Editor and the peer reviewers coming together and discussing their recommendations until a consensus is reached. When revisions are invited, a fact-based synthesis statement explaining their decision and outlining what is needed to prepare a revision will be listed below. The following reviewer(s) agreed to reveal their identity: Robert zeller.

Your study was reviewed by two experts and they found it to be an interesting report on the connectome of Ciona. Their reviews are given below. Please revise your manuscript to address their minor concerns.

Reviewer 1

The complete connectome of Ciona presents a valuable opportunity for dissecting neural circuits. Even more promising is the availability of tools for genetic and pharmacological perturbations, enabling researchers to evaluate the effects on behavior. This study leverages these advantages by mapping RNA expression patterns across the Ciona nervous system, using a combination of in situ hybridization (ISH) and imaging to determine the distribution of various glutamate receptor types. By employing MK801, an antagonist for NMDA receptors, they demonstrate that it completely inhibits the phototactic and dimming response in larvae. However, the study's reliance on RNA expression alone raises some concerns. The conclusions drawn from RNA expression may not fully reflect corresponding changes in protein expression. The subunit composition of AMPA and NMDA receptors significantly impacts their function, which could shed light on the puzzling finding that NMDAR blockade affects both phototaxis and the dimming response. While these initial studies are a crucial first step, further investigations into receptor composition, activity, and site of expression (synaptic vs extrasynaptic) would significantly enhance our understanding of functional connectivity of sensorimotor circuits.

EDITORIAL FEEDBACK: Please revise your Discussion to address this issue from Reviewer 1:

"The conclusions drawn from RNA expression may not fully reflect corresponding changes in protein expression. The subunit composition of AMPA and NMDA receptors significantly impacts their function, which could shed light on the puzzling finding that NMDAR blockade affects both phototaxis and the dimming response. While these initial studies are a crucial first step, further investigations into receptor composition, activity, and site of expression (synaptic vs extrasynaptic) would significantly enhance our understanding of functional connectivity of sensorimotor circuits."

Reviewer 2

This paper provides valuable gene expression mapping data for the Ciona nervous system. It demonstrates the predictability of neuronal circuit function based on the overlap of this expression data with the previously characterized connectome of the larva.

To date, the only chordate with a complete synaptic wiring diagram is the larva of the ascidian Ciona robusta. Compared to vertebrates, the simple Ciona larva is a tractable system for understanding brain function at the single cell level and has a few quantifiable behaviors that make such studies possible. Here the authors of this manuscript use whole mount fluorescent in situ hybridization (WMFISH) to examine glutamate receptor expression at the single cell level in the Ciona robusta larva. In Ciona, all of these neurons are sensory.

The authors examine the expression of the following glutamatergic ionotropic receptors (AMPA, NMDA and kainite) and the metabotropic receptor mGluR. Ciona has a single gene for each of these receptors. In this study, the authors generated 3D confocal stacks of the in situ data and used custom scripts to computationally map these expression patterns onto the connectome map generated by other research groups. The stereotypic arrangement of the nervous system cells made these mapping approaches possible.

With the above gene expression and mapping data in hand, the authors could corroborate previous studies such as a swimming response mediated by larval photoreceptors activity. Their analysis also suggests the epidermal sensory neurons (ESNs) may communicate with other ESNs, rather than interneurons, thus providing testable hypotheses for future experiments.

Lastly, the authors used a behavioral assay to test the hypothesis that NMDA receptors are required for sensorimotor responses. Previous studies had shown that interfering with AMPA-R function blocked negative phototaxis. Based on the in situ results, the authors predicted that the larval dimming response is mediated by NMDA-R and demonstrated this in behavioral assays thus demonstrating the predictability of neuronal circuit function based on mapped gene expression patterns.

Overall a nice paper demonstrating the power of combining gene expression at the single cell level with connectome mapping to place gene expression patterns in the context of neuronal connections.

Figure 1. Blue and orange arrowheads point to???

EDITORIAL FEEDBACK: Please revise Figure 1 and related text to address the last point made by the reviewer.
