# Supplementary figures and images for: Whole Nervous System Expression of Glutamate Receptors Reveals Distinct Receptor Roles in Sensorimotor Circuits
Source: eNeuro. 2024 Sep 18;11(9):ENEURO.0306-24.2024. doi: 10.1523/ENEURO.0306-24.2024 (PMC11419600; doi:10.1523/ENEURO.0306-24.2024)

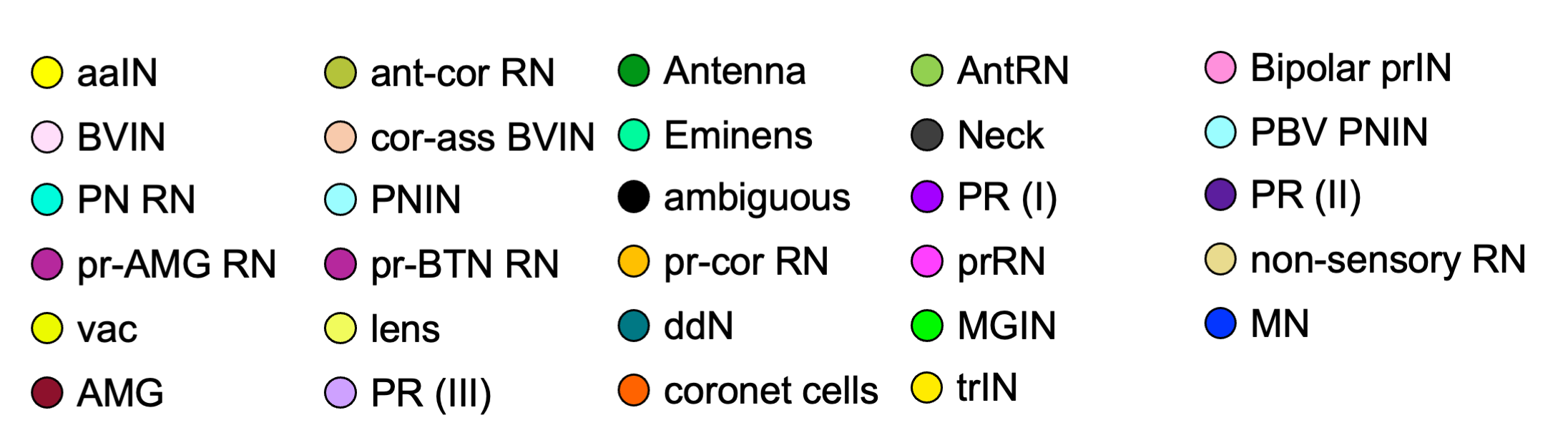

Supplement: Figure 4-1 — Key for color-coding of neuron classes shown in Figure 4B. Download Figure 4-1, TIF file. [file eneuro-11-ENEURO.0306-24.2024-s005.tif]

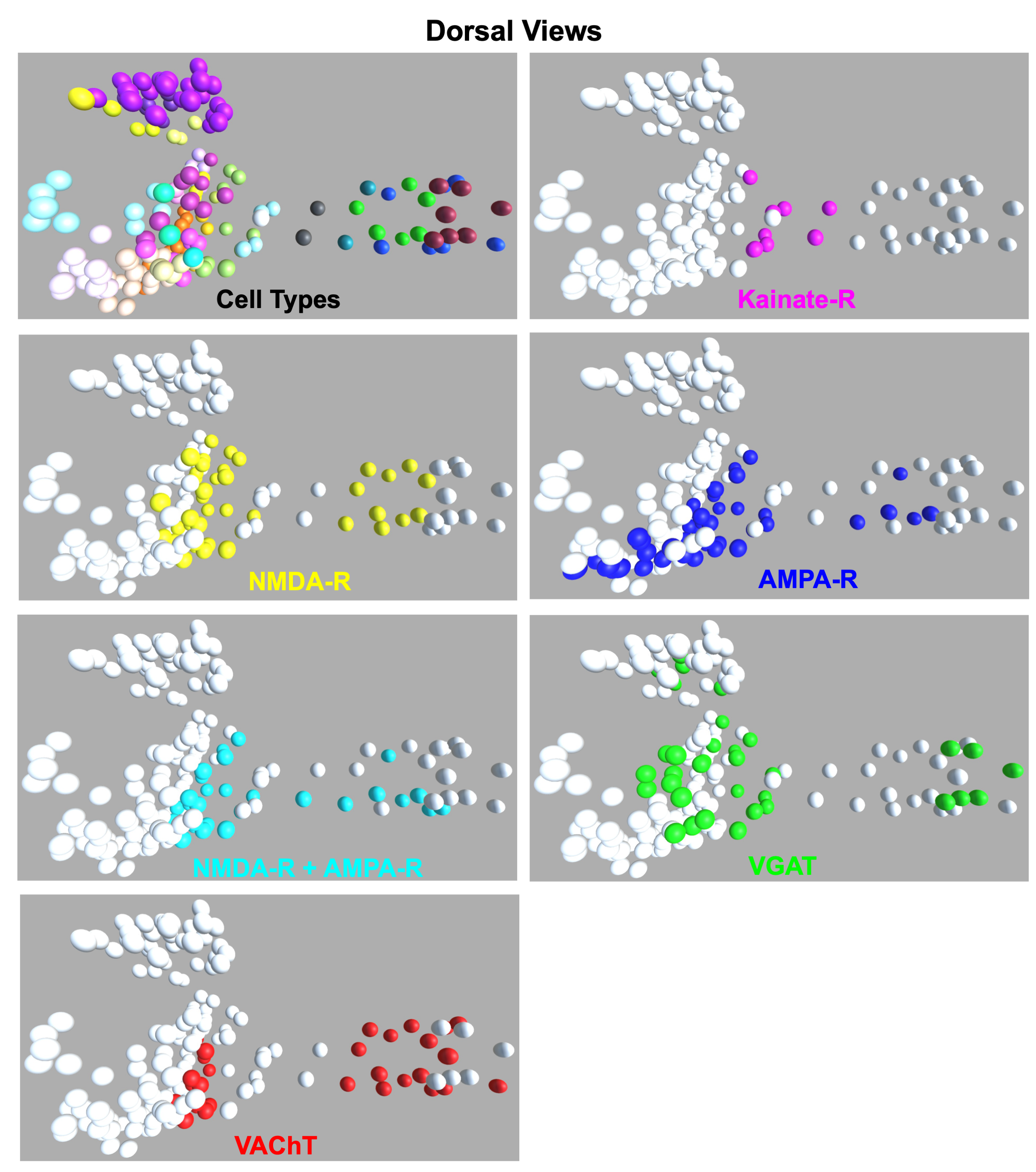

Supplement: Figure 4-2 — Dorsal Views of Figure 4, panels B-H. Download Figure 4-2, TIF file. [file eneuro-11-ENEURO.0306-24.2024-s006.tif]

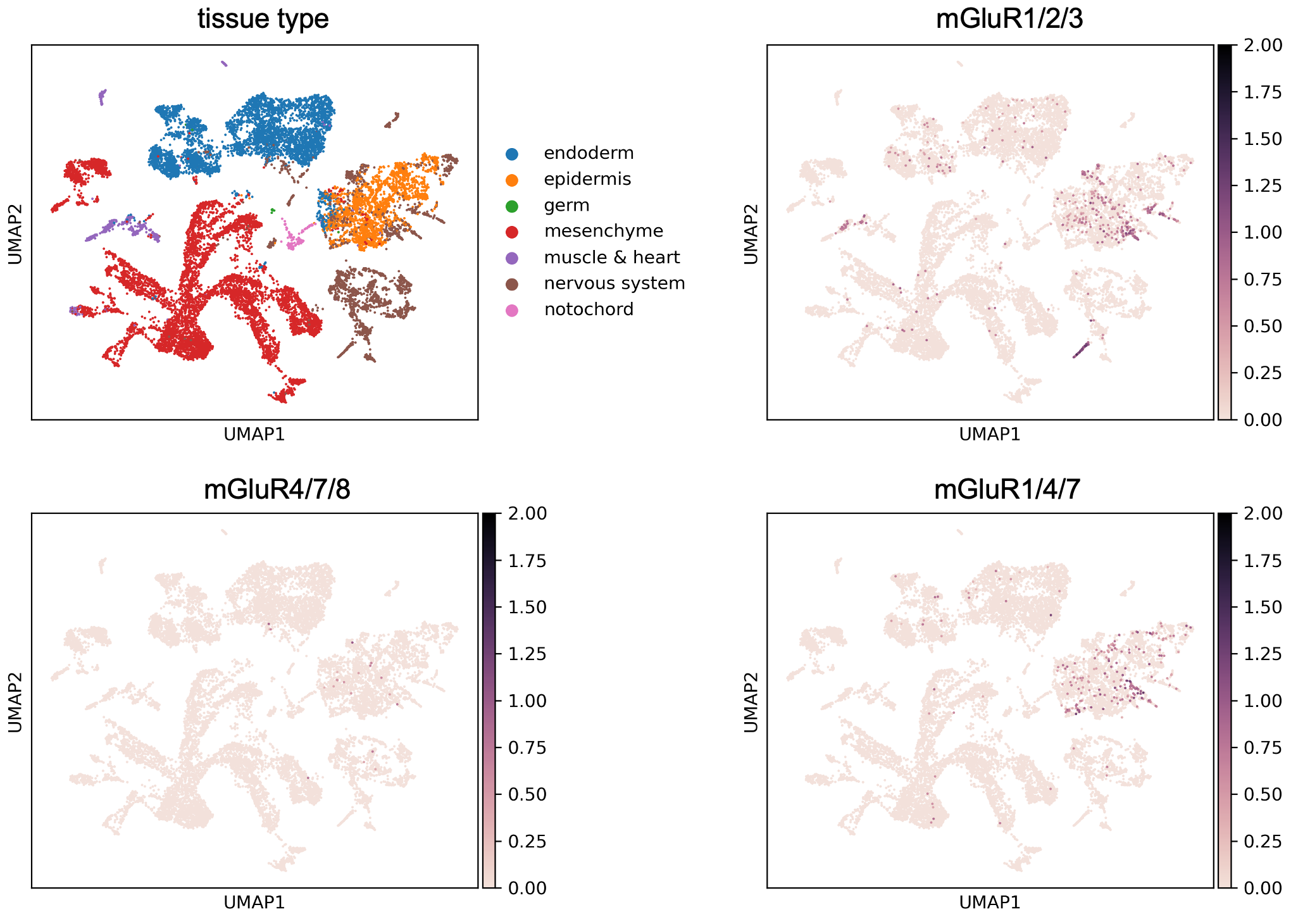

Supplement: Figure 5-1 — Expression of mGlu receptors (mGluR) in the Ciona larvae single cell RNaseq dataset from (Cao et al., 2019) . The top left panel shows cells clustered by UMAP analysis and color-coded by tissue type. The remaining three panels show the distribution of the three putative mGlu receptors in the clusters. Notice that mGluR123 is more highly expressed than the other two. Download Figure 5-1, TIF file. [file eneuro-11-ENEURO.0306-24.2024-s007.tif]
